# Complete Sequence Analysis of Grapevine Leafroll-Associated Virus 4 and Interactions Between the Encoded Proteins

**DOI:** 10.3390/v17070952

**Published:** 2025-07-05

**Authors:** Tingting Du, Yuxin Hao, Jie Gao, Shane Qiao, Guojun Hu, Fang Ren, Xudong Fan, Yafeng Dong

**Affiliations:** National Center for Eliminating Viruses from Deciduous Fruit Tree, Research Institute of Pomology of CAAS, Xingcheng 125100, China; 17352276700@163.com (T.D.); haoyuxin@caas.cn (Y.H.); g15689460301@163.com (J.G.); qse18835459568@163.com (S.Q.); huguojun@caas.cn (G.H.); renfang2015@163.com (F.R.)

**Keywords:** grapevine leafroll-associated virus 4, complete genome sequences, bimolecular fluorescence complementation, yeast two-hybrid, interactions between the proteins

## Abstract

Grapevine leafroll disease is one of the most devastating diseases in the global viticulture industry. Grapevine leafroll-associated virus 4 is one of the main pathogens causing this disease. In this study, we obtained the complete genome sequences of two Chinese isolates of GLRaV-4 from ‘Baisainie’ and ‘Fantasy Seedless’ grapevines through high-throughput sequencing and overlapping RT-PCR combined with RACE technology. The sequences contain 13,814 and 13,824 nucleotides and code six open reading frames, respectively. Phylogenetic trees based on the coat protein (CP) and heat shock protein 70 (HSP70) genes show that in addition to other GLRaV-4 strains (strains 5, 6, 9, Pr, and Car), the GLRaV-4 strains were divided into two distinct groups. The two isolates obtained in this study were classified into separate branches within GLRaV-4 Group 1. Additionally, we systematically investigated the interactions between the proteins encoded by GLRaV-4 using the yeast two-hybrid and bimolecular fluorescence complementation techniques. We found significant interactions between the GLRaV-4-encoded p23 and HSP70 and CP. This study first reports the complete genomes of two different GLRaV-4 isolates from China and suggests that p23 protein encoded by GLRaV-4 may play an important role in viral pathogenicity due to its interactions with the other two proteins.

## 1. Introduction

Grapevine (*Vitis* spp.) is regarded as one of the most widely grown fruit crops worldwide and is widely used to produce wine, sultanas, seed oils, and other by-products [1,2,3]. Among the various pathogens that infect grapevines, viruses are particularly damaging, with 102 known viruses currently infecting grapevines [4]. Grapevine leafroll disease (GLD), caused by grapevine leafroll-associated viruses (GLRaVs), is one of the most common diseases affecting all grapevine varieties (including those used for juice wine, table grapes, and their rootstocks) [5].

GLD is a widely occurring viral disease in grapevines [6,7]. It has been distributed worldwide, including in Europe, South America, North America, the Middle East, and North and Southern Africa. GLD occurs almost wherever wine grapes are cultivated [8,9]. Previous studies have shown that GLD has an adverse impact on the vine vigor, yield, and grape quality in several countries [10,11,12]. In more heavily infected parks, crop yields of 40–60% have been lost [13]. In particular, in red-fruited cultivars, the color of the berries was poor due to virus infection, which can cause severe economic losses throughout the life cycle of the vineyard [7]. The symptoms of GLD may vary depending on the virus variant, grape variety, rootstock type, and the grape’s growing environment. For example, American rootstocks usually have no symptoms, only decreased vigor [9,14]. The symptoms of GLD differ between the white and red varieties, although both exhibit leaf rolling. In the white variety, the leaves show only yellowing. In contrast, in the red variety, the symptoms are more visible, with a purplish-red coloration in the area between the veins, but the veins remain green [6]. The main pathogens of GLD are GLRaVs. The GLRaVs belong to the *Closteroviridae* family, with grapevine leafroll-associated virus 1, 3, 4, and 13 (GLRaV-1, GLRaV-3, GLRaV-4, and GLRaV-13) belonging to the genus *Ampelovirus*, grapevine leafroll-associated virus 2 (GLRaV-2) belonging to the genus *Closterovirus*, and grapevine leafroll-associated virus 7 (GLRaV-7) belonging to genus *Velarivirus*. In previous studies, grapevine leafroll-associated viruses 5 to 6, 9, De, Pr, and Car (GLRaV-5 to 6, 9, De, Pr, and Car) were categorized as different strains of GLRaV-4 based on their biological, epidemiological, and serological characteristics [15]. The genomes of these viruses were all positive-sense single-stranded RNAs.

GLRaV-4 belongs to the genus *Ampelovirus*. GLRaVs of the genus *Ampelovirus* are divided into two subgroups based on their phylogenetic differences, genome size, and organization [16,17,18]. The GLRaV-1, -3, and -13 genomes vary in size between ~18.5 and ~18.9 kilobases (kb), encode 9 to 12 ORFs, and are clustered under subgroup I. In contrast, GLRaV-4 and its strains with smaller genome sizes (between ~ 13.6 and ~ 13.8 kb and encoding six ORFs were assigned to subgroup II. Interestingly, the currently known GLRaV-4 strains, unlike other GLRaVs, lack a minor coat protein (CPm) homolog, and the viral particles appear to be entirely covered by CP [18]. GLRaV-4 is unique in that it consists of several strains, designated as GLRaV-4 strains -4, -5, -6, -9, -Pr, and -Car. Studies have shown that GLRaV-4 is primarily transmitted through grafting and vector insects, with the latter playing a crucial role in viral spread, primarily facilitated by mealybugs and soft-scale insects [19]. Previous reports have demonstrated high variability in the GLRaV-4 CP sequence [20]. Several GLRaV-4 strains trigger mild to moderate leaf curl symptoms in red-berried cultivars of *Vitis vinifera*, which were evident in infected field samples and indicator plants [19,21,22,23].

However, no validated whole-genome sequence of GLRaV-4 has been reported in China. Additionally, there is a relative lack of research on the interactions between GLRaV-4-encoded proteins. This deficiency somewhat limits our in-depth understanding of the genetic diversity and pathogenic mechanisms of GLRaV-4. In light of this, this study focused on the genomics and molecular interaction mechanisms of GLRaV-4. By employing high-throughput sequencing (HTS) technology, we successfully validated and obtained two complete GLRaV-4 genome sequences, which represent the first report of the whole-genome sequence of GLRaV-4 from China and provided critical foundational data for subsequent virus detection, classification, and evolutionary studies. Furthermore, we systematically studied the interactions between the proteins encoded by GLRaV-4 using yeast two-hybrid (Y2H) and bimolecular fluorescence complementation (BIFC) technologies.

## 2. Materials and Methods

### 2.1. Plant Material

The experimental materials were taken from the National Center for Eliminating Viruses material preservation garden from a deciduous fruit tree, Research Institute of Pomology of Chinese Academy of Agriculture Sciences in Xingcheng city, Liaoning province. Leaves of ‘Baisainie’ grapevine with rolled leaves and yellow spots and ‘Fantasy Seedless’ grapevine with rolled leaves and red spots were frozen in liquid nitrogen and sent to Biomarker Biology Technology (Beijing, China) for RNA-seq. *Nicotiana benthamiana* plants were incubated for 4–6 weeks at 20–25 °C for 16 h in light treatment and 8 h in dark treatment. Specific procedures for virus inoculation are described in Ref. [24]. After inoculation, plants maintained the same growth conditions for 48–96 h.

### 2.2. RNA-Seq

Leaf samples from ‘Baisainie’ and ‘Fantasy Seedless’ were used to extract total RNA. Ribosomal RNA was removed from total RNA extracts using the Epicentre Ribo-Zero rRNA Removal Kit (Epicentre, Madison, WI, USA). The ribosomal RNA-depleted RNA sample was then used to construct a cDNA library using a TruSeq RNA Sample Prep Kit (Illumina, San Diego, CA, USA), which was sequenced on an Illumina HiSeq 4000 platform with a paired-end 150 bp setup (Biomarker Biology Technology, Beijing, China). Reads mapping to the grapevine genome (PN40024 assembly 12X) were filtered out by hierarchical indexing using hisat2 software [25]. Unmapped reads were used for de novo assembly and Blast analysis embedded in VirusDetect5.10.0 (http://virusdetect.feilab.net/cgi-bin/virusdetect/index.cgi, accessed on 12 February 2025) [26].

### 2.3. Amplification of GLRaV-4 and Sequencing Analysis

RNA extraction and reverse transcription were carried out using a column extraction method, as described in the literature [14,27]. Primer pairs were designed to amplify the GLRaV-4 genome and validate high-throughput sequencing results by overlapping region sequences based on RNA-Seq results (Tables S1 and S2). The 20 fragments amplified by PCR were recovered and purified by Aidlab Agarose Gel Purification Recovery Kit (Aidlab, Beijing, China) and ligated into *E. coli* DH5α receptor cells (Figure S1). Afterward, three positive clones were taken for sequencing, and consensus sequences were obtained when three independent clones had ≥98% homology. The 5′ and 3′ untranslated regions (UTRs) were subsequently amplified using the rapid amplification of cDNA ends (RACE) strategy employing the SMARTer^®^ RACE 5′/3′ Kit (TaKaRa) to ensure the accuracy of the assembled GLRaV-4 genome sequence. The sequences of the overlapping fragments were assembled using Geneious Prime2024.0 software [28].

The ORF Finder tool from the National Center for Biotechnology Information (NCBI) was used to identify potential ORFs in the genomic RNA, and the CD-search tool was employed to detect conserved domains (https://www.ncbi.nlm.nih.gov/Structure/cdd/wrpsb.cgi, accessed on 12 February 2025). Subsequently, MEGA11 software was utilized to compare the CP and HSP70 gene sequences obtained in this study with the corresponding full-length sequences registered in GenBank [29]. Phylogenetic analysis was conducted to construct a Neighbor-Joining (NJ) evolutionary tree to reveal the evolutionary relationships of GLRaV-4 globally. Recombination events were analyzed using SBP (single breakpoint scanning) and GARD (genetic algorithm recombination) in Datamonkey2.0 software [30].

### 2.4. Construction of Vectors Containing Candidate Genes

The vector used in this study was constructed by primers in Table S3, according to the previously described method [24]. In constructing the vectors used in the Y2H system, the vectors pGADT7 and pGBKT7 were linearized using primer pairs AD-F/R and BD-F/R. Primer pairs AD-RdRp-F/R, AD-p5-F/R, AD-HSP70-F/R, AD-p60-F/R, AD-CP-F/R, AD-p23-F/R, BD-RdRp-F/R, BD-p5-F/R, BD-HSP70-F/R, BD-p60-F/R, BD-CP-F/R, and BD-p23-F/R were used to amplify fragments of the target genes to construct the six prey proteins and decoy proteins, respectively. The correctly sequenced plasmids were transferred into the Y2H competent cells. In BIFC experiments, PCR linearization of the vectors pEarleyGate202-YC and pEarleyGate201-YN was carried out using primer pairs cYFP-F/R and nYFP-F/R, respectively. Primer pairs nYFP3-F/R, cYFP3-F/R, nYFP5-F/R, cYFP5-F/R, nYFP6-F/R, and cYFP6-F/R were used to amplify fragments of the HSP70, CP, and p23 proteins, respectively, that have reciprocal interactions. These vectors were sequenced and placed at −20 °C for subsequent transformation into *Agrobacterium* GV3101.

### 2.5. Y2H Assay and BIFC Assay

Yeast small-scale transformation was performed according to the manufacturer’s protocol (Coolaber, CC309, Beijing, China). The bait vectors were separately transformed into Y2HGold competent cells, with the pGBKT7 vector serving as a positive control. The transformants were plated on SD-Trp dropout medium to assess whether the target protein affects yeast growth. The bait vector and the pGADT7 empty vector were co-transformed into Y2HGold competent yeast cells and plated on dropout media SD/-Leu-Trp and SD/-Leu-Trp-Ade-His +X to check whether the target protein displayed self-activation; the bait proteins and prey proteins were introduced into Y2HGold yeast competent cells pairwise and plated on dropout media SD/-Leu-Trp and SD/-Leu-Trp-Ade-His +X to screen for interacting proteins, in which pGBKT7-P53 + pGADT7-T and pGBKT7-lam+pGADT7-T were used as positive and negative controls, respectively. Subsequently, the positive clones were screened, and the positive clones were cultured to an OD600 of 0.8, and diluted with ultrapure water at a concentration gradient of 10^0^, 10^−1^, 10^−2^, 10^−3^, and 10^−4^, respectively. These strains and the control strains were spot-cultivated on different media to check the growth and color change. Each experiment was repeated three times.

In BIFC experiments, *Agrobacterium* rhizogenes strains GV3101 carrying pEarleyGate201-YN-HSP70, pEarleyGate202-YC-p23, pEarleyGate201-YN-p23, pEarleyGate202-YC-HSP70, pEarleyGate201-YN-CP, pEarleyGate202-YC-CP, pEarleyGate201-YN-A BI5, and p19 were cultured separately, with pEarleyGate202-YC-p53/pEarleyGate201-YN-ABI5 as a negative control. After incubation, *Agrobacterium* was resuspended in the infiltration solution (10 mmol/L MgCl_2_, 10 mmol/L MES, 200 µmol/L acetosyringone) to an OD600 of 1. Each pair of screened proteins was injected in equal volume into 4–5-week-old *N. benthamiana* plants. Fluorescence in the injected area of the *N. benthamiana* was observed after 48–72 h of incubation in darkness, and each experiment was repeated three times.

## 3. Results

### 3.1. HTS Data Analysis and Validation of High-Throughput Sequencing Results

We found nine different viruses and three viroids from the two grapevines. The identified viruses cover members of several genera, as follows: *Vitivirus* (grapevine virus B, GVB); *Ampelovirus* (GLRaV-4); *Closterovirus* (GLRaV-2); *Foveavirus* (grapevine rupestris stem pitting-associated virus, GRSPaV); *Marafivirus* (grapevine rupestris vein feathering virus, GRVFV); grapevine asteroid mosaic-associated virus (GAMaV); grapevine Syrah virus 1 (GSyV-1); grapevine fleck virus (GFkV), and *Trichovirus* (grapevine pinot gris virus, GPGV). The viroids’ genera included *Apscaviroid* (grapevine yellow speckle viroid 1 and Grapevine yellow speckle viroid 2, GYSVd1 and GYSVd2) and *Hostuviroid* (hop stunt viroid, HSVd). The “Baisainie” sample contained GLRaV-4, GRSPaV, GFkV, GPGV, GAMaV, GSyV-1, GYSVd1, and HSVd. The “Fantasy Seedless” sample contained GLRaV-4, GLRaV-2, GRSPaV, GFkV, GPGV, GVB, GRVFV, GYSVd1, GYSVd2, and HSVd. Table S4 shows the depth, contigs, and coverage based on the high-throughput sequencing results and bioinformatics analysis. In terms of the contig group statistics, GLRaV-4 had 1 contig in the “Baisainie” sample, covering 99.1% of the reference genome (KY821095), with a depth of 56.6, and 25 contigs in the “Fantasy Seedless” sample, covering 100% of the reference genome (KY821095), with a depth of 472.6 (Table S4).

To further validate the high-throughput sequencing results, two samples were subjected to RT-PCR using primers specific for GLRaV4, GLRaV2, GRSPaV, GRVFV, GPGV, GFKV, GSyV-1, GAMaV, GVB, GYSVd-1, GYSVd-2, and HSVd (Table S1). The subsequent purification, recovery, and sequencing of the PCR products showed that the nucleic acid sequences of the viruses in the samples matched those of the target viruses. The RT-PCR results were found to be consistent with the high-throughput sequencing results.

### 3.2. Genome Sequence and Phylogenetic Analysis

**Figure 1:**
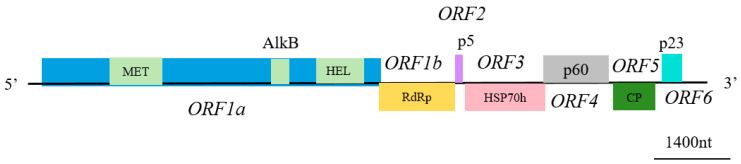
Schematic diagram of the complete genome structure of GLRaV-4.

The complete genome sequences of two GLRaV-4 isolates were obtained, named BSN and FaS. The ORF Finder tool from NCBI was used to identify the potential ORFs. We conducted a detailed analysis of the genomic structure, with the results shown in Figure 1. Both isolates exhibited the typical characteristics of single-stranded RNA viruses, with the genome containing six conserved ORFs (ORF1-ORF6) arranged in an order highly consistent with other members of the genus *Ampelovirus*. The full lengths of the isolates BSN and FaS in this study were 13,814 and 13,824 nt, respectively. The length differences primarily originated from polymorphic variation in the non-translated regions at the ends of the genome. The two complete GLRaV-4 genomes have been submitted to the GenBank database under accession numbers PV540235 and PV540236, respectively.

To thoroughly investigate the genetic characteristics of GLRaV-4 and its diversity, we selected representative GLRaV-4 genome sequences from the GenBank database, which were chosen based on their wide geographic distribution and phylogenetic diversity to ensure comprehensive coverage of the genetic variation spectrum of GLRaV-4. Subsequent systematic comparisons were made with the nucleotide and amino acid sequences of the two GLRaV-4 isolates, BSN and FaS, obtained in this study (Tables S5 and S6). The results showed that the 5′ UTR showed significant genetic divergence among the different GLRaV-4 isolates, with isolates BSN and FaS showing 40–70% homology with most of the isolates and up to 85–97% homology with only a few isolates. In the 3′ UTR, isolates BSN and FaS showed 54.6–95.3% and 52.2–100% nucleotide similarity with all of the reported GLRaV-4 isolates, respectively. In ORF2–ORF6, the nucleotide and amino acid sequence similarity of isolate BSN with other known GLRaV-4 isolates was 64.5–87.9%/69.6–89.1%, 61.43–89.1%/67.2–93.6%, 59.3–90.1%/63.6–94.6%, 66.9–89.7%/73.9–93.8% and 58.3–92.8%/53.1–91.3%, respectively. Compared to the isolate BSN, the FaS isolate exhibited higher nucleotide and amino acid sequence similarity to other known GLRaV-4 isolates in the ORF2–ORF6 regions of 66.7–100%/73.9–100%, 61.3–93.3%/67.5–99.8%, 59.4–99.6%/61.1–100%, 67.5–99.5%/73.2–98.9%, and 59.1–99.7%/53.14–99.0%. Genome-wide nucleotide-based comparisons revealed that both isolates BSN and FaS showed the highest homology, with the Pakistani isolate LH3 (KY821095) at 86.8% and 99.2%, respectively.

**Figure 2:**
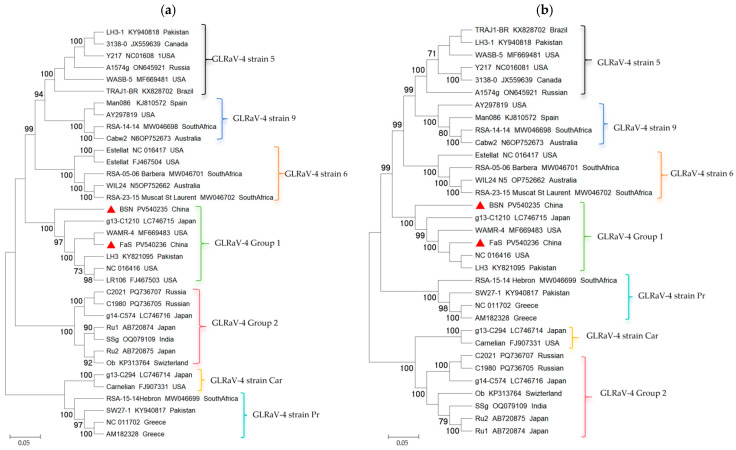
**Phylogenetic analysis of GLRaV-4 isolates based on the sequences of CP and HSP70 gene**. Corresponding sequences provided by GenBank are identified with the accession number and origins, and the numbers on the nodes indicate the bootstrap support (1000 replicates). Values below 70% are not shown. (**a**) Phylogenetic tree constructed based on the GLRaV-4 CP gene. (**b**) Phylogenetic tree constructed based on the GLRaV-4 HSP70 gene. Sequences obtained in this study are denoted by red triangles.

The sequenced CP and HSP70 gene sequences were subjected to phylogenetic tree analysis, along with the CP and HSP70 gene sequences of GLRaV-4 isolates and several other strains (5, 6, 9, Pr, and Car strains) (Figure 2). The results showed that the evolutionary tree analysis of the CP gene was consistent with that of the HSP70 gene, and that except for a few strains (strains 5, 6, 9, Pr, and Car), the GLRaV-4 variants could be divided into two separate groups. The isolates of BSN and FaS obtained in this study were classified into different branches of GLRaV-4 group 1, which also included isolates from Pakistan, the USA, Japan, etc.; GLRaV-4 group 2 contained isolates from the USA, India, Russian, Nyon, Pakistan, and Japan. Isolate BSN is more closely related to the Pakistani isolate KY821095, with an amino acid homology of 93% for both HSP70 and CP. In contrast, the isolate FaS was closer to the USA isolate NC_016416, with nearly 99% amino acid homology for HSP70 and CP.

The genetic distances between the GLRaV-4 CP and HSP70 gene variant groups are shown in Table 1 and Table 2. The largest genetic distance within the CP variant group was found in GLRaV-4 strain Car (0.224 ± 0.015), followed by GLRaV-4 Group 2 (0.115 ± 0.007), GLRaV-4 strain 6 (0.078 ± 0.007), GLRaV-4 strain Pr (0.063 ± 0.006), GLRaV-4 Group 1 (0.048 ± 0.004), and GLRaV-4 strain 5 (0.044 ± 0.004), and the smallest genetic distance was in GLRaV-4 strain 9 (0.027 ± 0.004). The genetic distances between the variant groups ranged from 0.184 ± 0.013 to 0.306 ± 0.015 (Table 1). The largest genetic distance within the HSP70 variant group was in GLRaV-4 strain Car (0.260 ± 0.011), followed by GLRaV-4 Group 2 (0.118 ± 0.005), GLRaV-4 strain 6 (0.093 ± 0.005), GLRaV-4 strain Pr (0.079± 0.004), GLRaV-4 Group 1 (0.062 ± 0.003), and GLRaV-4 strain 5 (0.056 ± 0.003), and the smallest genetic distance was in GLRaV-4 strain 9 (0.039 ± 0.003). The genetic distances between the variant groups ranged from 0.240 ± 0.010 to 0.384 ± 0.010 (Table 2). Thus, the genetic distances between the seven groups were greater than the genetic distances within each group. This supports the fact that the GLRaV-4 isolates were divided into seven groups based on the CP and HSP70 genes.

### 3.3. Interactions Between Proteins Encoded by GLRaV-4

**Figure 3:**
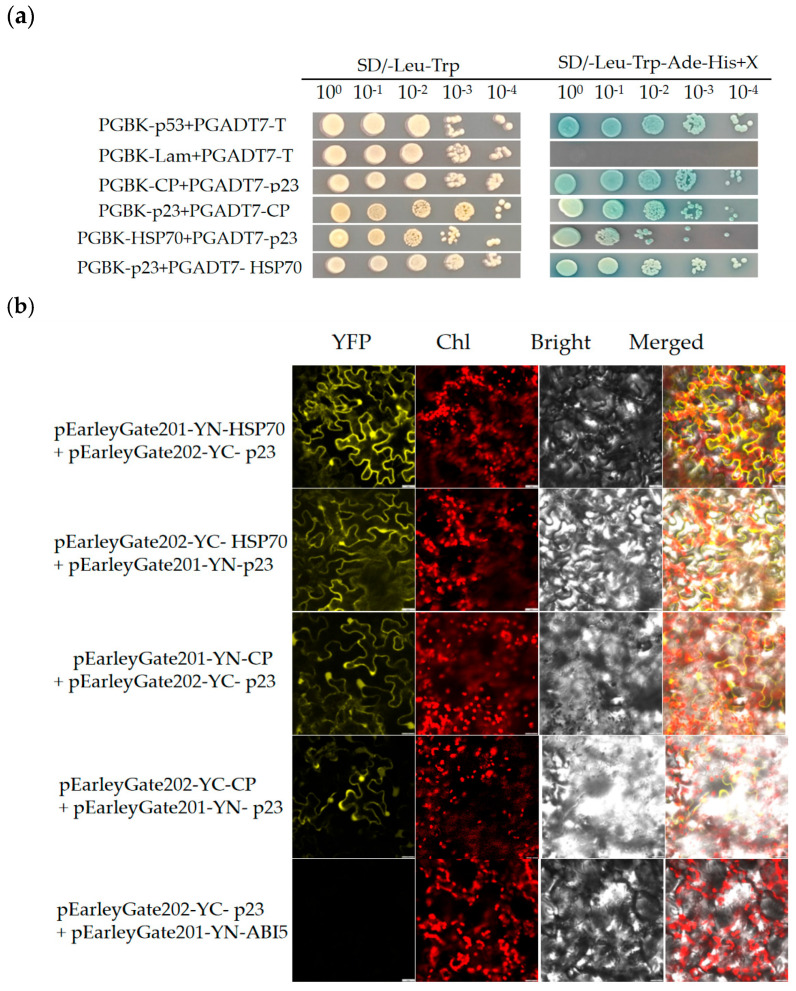
**p23 protein interacts with CP and HSP70 protein in vitro and in vivo**. (**a**) The interaction of p23 with CP and HSP70 was determined by Y2H in yeast cells. Yeast cells carrying the corresponding plasmids were subjected to a 10-fold gradient dilution, and in SD/-Leu-Trp and SD/-Leu-Trp-Ade-His +X medium for 2–3 days. Yeast cells co-transformed with PGBK-p53 and PGADT7-T were used as positive controls, and yeast cells co-transformed with PGBK-Lam and PGADT7-T were used as negative controls. (**b**) BiFC analysis of the interaction of p23 with CP and HSP70 in leaves of *N. benthamiana*. The pEarleyGate202-YC- p23 + pEarleyGate201-YN-ABI5 was used as a negative control. YFP indicates target protein fluorescence channel; Chl indicates chloroplast autofluorescence channel; Bright indicates bright field channel; Merge indicates superimposed channel of YFP, Chl, and Bright. Scale bar = 25 μm. All experiments were performed in three independent biological replicates with consistent results.

In this study, we constructed a decoy vector (pGBKT7-BD) and a prey vector (pGADT7-AD) containing ORFs of GLRaV-4. The Y2H assay verified the interactions between the GLRaV-4-encoded proteins. Firstly, the toxicity assay results showed that the growth of yeast strains containing the decoy vectors was consistent with that of yeast containing the pGBKT7 empty vector (Figure S2), indicating that the target protein had no toxic effect on the growth of yeast cells. Secondly, in the self-activation assay, the bait vector was co-transformed with the pGADT7 empty vector into yeast Y2HGold receptor cells. The positive clones were all able to grow normally on the two-deficient medium (SD/-Leu-Trp). In contrast, on the four-deficient medium (SD/-Leu-Trp-Ade-His), only the positive control (pGBKT7-p53+pGADT7-T) was able to grow. Blue staining appeared on the medium containing the X-α-gal on four-deficient medium (SD/-Leu-Trp-Ade-His +X), showing blue colored spots (Figure S3). The negative control (pGBKT7-lam + pGADT7-T) and the experimental group (bait protein + pGADT7) grew on SD/-Leu-Trp medium, but failed to grow on SD/-Leu-Trp-Ade-His medium, suggesting that the bait proteins do not have self-activating activity and can be used in subsequent screening experiments for interacting. We performed one-to-one mating experiments of each bait plasmid with the prey plasmids, and the results showed that only the combinations of pGBK-CP + pGADT7-p23 and pGBK-HSP70 + pGADT7-p23 grew normally on both SD/-Leu-Trp medium and SD/-Leu-Trp-Ade-His medium and formed blue colonies on SD/-Leu-Trp-Ade-His +X medium (Figure 3a). This indicates that the CP and HSP70 proteins encoded by GLRaV-4 interact with the p23 protein.

The Y2H technique can only detect interacting proteins in live yeast cells and is unable to verify protein interactions within the plant body [31,32]. The advantage of the BiFC method over other complementary methods is the strong intrinsic fluorescence of the assembled complexes, which allows for direct observation of protein interactions. Thus, with the BiFC method, the long-term observation of living cells is possible and the possibility of experimental manipulation altering the results is minimized [33]. To verify the results of the Y2H assay, we also used the BIFC assay in vivo to confirm the interaction of CP and HSP70 with p23 in plants, respectively. The pre-constructed *Agrobacterium* rhizogenes GV3101 carrying pEarleyGate201-YN-HSP70 and pEarleyGate201-YN-CP, and the *Agrobacterium* rhizogenes GV3101 cells carrying pEarleyGate202-YC- p23, respectively, were co-infiltrated and transformed into *N. benthamiana* leaves, and at the same time, the combination of a plasmid and pEarleyGate202-YC-p23/pEarleyGate201-YN-ABI5 co-infiltrated with *N. benthamiana* was used as a negative control. Three days after infiltration, the YFP fluorescence expression of the test samples was observed under a confocal laser scanning microscope (LEICA TCS SP8, German). By observational analysis, it was found that both pEarleyGate201-YN-HSP70/pEarleyGate202-YC-p23, pEarleyGate202-YC-HSP70/pEarleyGate201-YN-p23, pEarleyGate201-YN-CP/pEarleyGate202-YC-p23 showed fluorescence, while no fluorescence was observed in the negative control (Figure 3b), and the experiment was repeated at least three times. These results further confirmed that the GLRaV-4-encoded p23 protein was able to interact with HSP70 and CP.

## 4. Discussion

GLD is the most significant viral disease in global grape production. The way that grape plants reproduce and the frequent exchange of their propagation material facilitates the pathogen’s spread, leading to even more destructive leafroll diseases [34]. The previous study has already demonstrated that GLRaV-4 may induce leafroll symptoms in grapevines [24], and perhaps for this reason, GLRaV-4 has not been included in an unregulated list of viruses with limited or no known harmful effects on vine vigor, yield, or fruit quality [4]. Currently, with the application of high-throughput sequencing, an increasing number of GLRaV-4 isolates have been reported from countries such as the United States, South Africa, Hungary, Italy, Chilean, India, Spain, and Japan. However, there have been no reports of GLRaV-4 isolates and their full-length genome sequences from China thus far. In the present study, the full-length genome sequences of two Chinese GLRaV-4 isolates were reported through the combination of high-throughput sequencing and Sanger sequencing, providing a basis for studying the gene function and pathogenicity of GLRaV-4.

Among the GLRaVs belonging to *Ampelovirus*, the 5′ UTRs of GLRaV-1, GLRaV-3, and GLRaV-13 exhibit significant length and sequence diversity. Specifically, the 5′ UTR length of these viruses varies between 672 and 1100 nt and is characterized, among other things, by including a variable number of repetitive sequences approximately 65 nt long [18,35]. In contrast, the 5′ UTR of the GLRaV-4 strain is relatively short, with a length of around 200 nt, and no similar sequence repeats were observed. In the present study, the 5′ UTR lengths of the isolates BSN and FaS were 214 nt and 215 nt, respectively, which are in approximate agreement with the 5′ UTR lengths of GLRaV-4 isolates reported in previous studies. Previous studies have demonstrated that the 5′ UTR sequence of GLRaV-3 encompasses key components essential for viral replication and that the 5′ UTR and 3′ UTR of Citrus tristeza virus (CTV) isolates form two stable stem-loop (SL) structures and a complex secondary structure consisting of up to 10 SLs, respectively, which are closely linked to viral replication and assembly [18]. The finding of this study reveal significant sequence divergence in the 5′ UTR of different GLRaV-4isolates, which may be linked to functional variations. Certain isolates possess distinct sequence features that could influence viral replication efficiency, host specificity, or interactions with other viral factors. These differences provide a vital foundation for in-depth research on the genomic structure and function of GLRaV-4, aiding in the comprehension of its biological characteristics.

The CP sequence variability of GLRaV-4 is relatively high; previous studies have differentiated GLRaV-4 strains into seven groups based on CP gene sequences, specifically strain 4, strain 5, strain 6, strain 9, strain Car, strain Pr, and strain Ob [20]. However, in this study, we combined the HSP70 and CP gene sequences of GLRaV-4 to reconstruct and group the phylogenetic tree of GLRaV-4 more carefully (Figure 2). Based on the significant genetic distance between Group 1 and Group 2 (Table 1 and Table 2), which was greater than the genetic distance between strain 5, strain 6, and strain 9, we renamed the seven groups as GLRaV-4 Group 1, GLRaV-4 Group 2, GLRaV-4 strain 5, GLRaV-4 strain 6, GLRaV-4 strain 9, GLRaV-4 strain Car, and GLRaV-4 strain Pr. At the same time, more diverse isolates were included within each branch, which enriched and improved the genetic evolutionary analysis system of GLRaV-4.

*Closteroviruses* encode a conserved quintuple gene block, which encodes the structural and movement proteins involved in the assembly and movement of viral particles. This block includes homologs of the HSP70 molecular chaperone and three divergent copies of the coat protein gene. The remaining genes exhibit significant variation in their number, function, and origin within and between *Closteroviruses* genera [36]. The 3′ proximal ORFs of members of the *Closteroviridae* family are not well conserved between genera and show no significant sequence or structural similarity [37]. In previous studies, it has been reported that GLRaV-3 p20B can interact with three of the four structural proteins of GLRaV-3, namely, HSP70h, CP, and CPm [38]. However, the results of this study show that the p23 protein encoded by GLRaV-4 can interact with both CP and HSP70 proteins. HSP70 is part of the head of the *Closterovirus* virion, and many viruses utilize the cellular HSP70 chaperone to assist in replication or viral particle assembly [39,40]. The interaction between the HSP70 homologous protein and the p23 protein may be critical for the replication and assembly of viral particles.

Members of the *Closterovirus* family have relatively low conservation of the proximal 3′ ORFs [37]. However, proteins encoded by similarly positioned 3′ ORFs in members of this virus family are involved in long-distance viral transport and inhibition of host RNA defense responses [41,42]. For example, HSP70 encoded by beet yellows virus (BYV) interacts with the p20 protein, which is required for long-distance virus transport through the phloem. At the same time, HSP70 establishes a molecular link between local and systemic transmission of plant viruses by docking long-distance transport factors to virus particles [42]. CP forms a long, helical body of the flexuous, filamentous virions, and functions as a major component of the tails of the short virion particles [42,43]. It was also found that both CTV p20 and CP interfered with the systematic propagation of silencing, whereas p23 could only inhibit localized silencing [44]. In addition to this, proteins such as p20 and p21 encoded by Pineapple Mealybug Wilt-Associated Virus 2 (PMWaV-2), p24 encoded by GLRaV-2, and p24 encoded by GLRaV-1 have been identified as RNA silencing suppressors (RSS) and have been shown to form homodimers [45,46]. In a previous study, the GLRaV-3-encoded silencing repressor p20B interacted with HSP70h, suggesting a similar morphology of the GLRaV-3 virion head [38]. At present, the mechanism of p23 in GLRaV-4 remains incompletely understood. Therefore, a comprehensive and in-depth investigation of the role of p23 in the GLRaV-4 infection cycle is essential for elucidating the mechanisms underlying GLRaV-4 viral replication as well as the assembly and structure formation of viral particles. This will help to analyze the origins and evolution of viruses belonging to the *Closterovirus* family.

The results of this study provide new insights into the mechanism of viral particle assembly and propagation of GLRaV-4, providing a basis for future research on the molecular mechanisms and development of prevention and control strategies. Unlike other GLRaV strains, the GLRaV-4 strain lacks the minor capsid protein (CPm) homolog, and the viral particles appear completely covered by CP [18]. We hypothesize that the p23 protein encoded by GLRaV-4 interacts with CPs and may be critical for maintaining the morphology and integrity of viral particles. These interactions may play a key role in viral replication, assembly, and propagation, and may be closely related to the pathogenesis of GLD. However, further studies are required.

## Figures and Tables

**Table 1:** Genetic distances between and within variant groups of GLRaV-4 CP genes.

Phylogroup		Between Groups				Within Groups	
Group 1							0.048 ± 0.004
Group 2	0.282 ± 0.011						0.115 ± 0.007
Strain 5	0.262 ± 0.010	0.294 ± 0.014					0.044 ± 0.004
Strain 6	0.270 ± 0.010	0.289 ± 0.014	0.223 ± 0.013				0.078 ± 0.007
Strain 9	0.271 ± 0.010	0.304 ± 0.015	0.184 ± 0.013	0.216 ± 0.013			0.027 ± 0.004
Strain Car	0.291 ± 0.015	0.310 ± 0.014	0.286 ± 0.014	0.284 ± 0.014	0.295 ± 0.014		0.224 ± 0.015
Strain Pr	0.284 ± 0.015	0.306 ± 0.015	0.289 ± 0.015	0.291 ± 0.015	0.300 ± 0.016	0.301 ± 0.015	0.063 ± 0.006

**Table 2:** Genetic distances between and within variant groups of GLRaV-4 HSP70 genes.

Phylogroup		Between Groups				Within Groups	
Group 1							0.062 ± 0.003
Group 2	0.357 ± 0.014						0.118 ± 0.005
Strain 5	0.265 ± 0.015	0.358 ± 0.011					0.056 ± 0.003
Strain 6	0.258 ± 0.014	0.364 ± 0.010	0.240 ± 0.010				0.093 ± 0.005
Strain 9	0.259 ± 0.015	0.356 ± 0.011	0.209 ± 0.010	0.240 ± 0.010			0.039 ± 0.003
Strain Car	0.383 ± 0.010	0.364 ± 0.010	0.378 ± 0.011	0.372 ± 0.010	0.378 ± 0.010		0.260 ± 0.011
Strain Pr	0.311 ± 0.011	0.371 ± 0.011	0.311 ± 0.011	0.310 ± 0.010	0.308 ± 0.011	0.384 ± 0.010	0.079 ± 0.004

## Data Availability

Data presented in the study are included in the article/Supplementary Material; further inquiries may be directed to the corresponding author.

## Supplementary Materials

The following supporting information can be downloaded at https://www.mdpi.com/article/10.3390/v17070952/s1: Figure S1. PCR amplification results of the whole-genome sequence GLRaV-4. a: Amplification of the full sequence of the BSN isolate using the primer pair BSN-GLRaV-4-1F/1R-10F/10R. b: Amplification of the full sequence of FaS isolate using primer pairs FaS-GLRaV-4-1F/1R-10F/10R; Figure S2: Yeast cells carrying the bait plasmids HSP70, cp, and p23 were inoculated in SD/-Trp medium for 2–3 days; Figure S3: Plasmids HSP70, cp, and p23 were co-transformed with PGADT7-T in yeast cells Y2H in a 10-fold gradient dilution and inoculated in SD/-Leu-Trp +X and SD/-Leu-Trp-Ade-His +X medium for 2–3 days; Table S1: The primers for PCR amplification of the whole-genome sequence of GLRaV-4 isolates BSN and FaS; Table S2: The primers used in virus detection to validate the high-throughput sequencing results; Table S3: The primers used to construct the vectors; Table S4: Virus species analyses of high-throughput sequencing data; Table S5: Percentage of nucleotide (nt) or amino acid (aa) identity between the genomes/genome products of BSN and other GLRaV-4 isolates; Table S6: Percentage of nucleotide (nt) or amino acid (aa) identity between the genomes/genome products of isolate FaS and other GLRaV-4 isolates.
