# Supplementary material for: Complete Sequence Analysis of Grapevine Leafroll-Associated Virus 4 and Interactions Between the Encoded Proteins
Source: Viruses. 2025 Jul 5;17(7):952. doi: 10.3390/v17070952 (PMC12299434; doi:10.3390/v17070952)
Supplement: Supplementary file 1 [file viruses-17-00952-s001.zip › Supplementary figures 1 and 3.pdf]

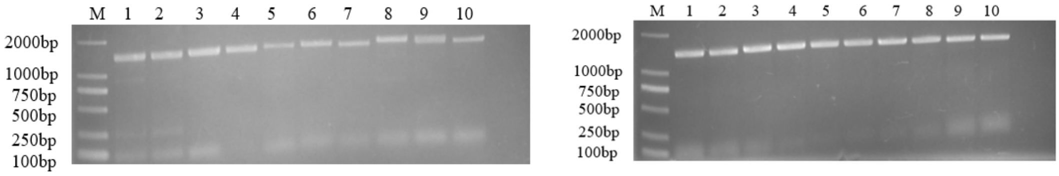

Figure S1. PCR amplification of the whole genome sequence of GLRaV-4. a：The full sequence of the BSN isolate was amplified using the primer pair BSN-GLRaV-4-1F/1R- 10F/10R. b：Amplification of the full sequence of FaS isolate using primer pairs FaS-GLRaV-4-1F/1R- 10F/10R.

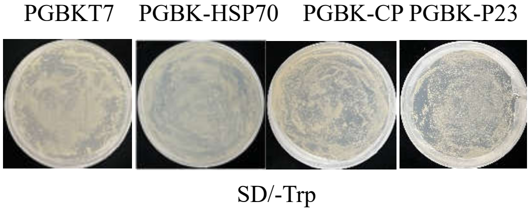

Figure S2. Yeast cells carrying the bait plasmids HSP70, cp and p23 were inoculated in SD/-Trp medium for 2-3 days. PGBKT7-transformed yeast cells as a positive control.

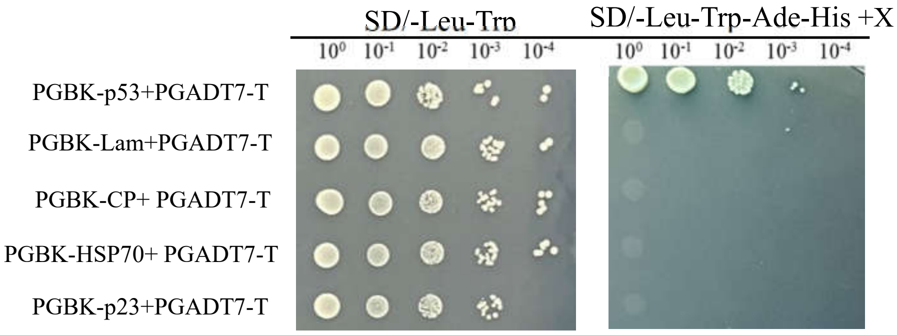

Figure S3. Plasmids HSP70, cp and p23 were co-transformed with PGADT7-T in yeast cells Y2H in a 10-fold gradient dilution and inoculated in SD/-Leu-Trp +X and SD/-Leu-Trp-Ade-His +X medium for 2-3 days. yeast cells co-transfected with PGBK-p53 and PGADT7-T were used as a positive control; yeast cells co-transfected with PGBK-Lam and PGADT7-T were used as a negative control. PGBK-P53 and PGADT7-T co-transformed yeast cells were used as positive control; PGBK-Lam and PGADT7-T co-transformed yeast cells were used as negative control.
