# Supplementary material for: Complete Sequence Analysis of Grapevine Leafroll-Associated Virus 4 and Interactions Between the Encoded Proteins
Source: Viruses. 2025 Jul 5;17(7):952. doi: 10.3390/v17070952 (PMC12299434; doi:10.3390/v17070952)
Supplement: Supplementary file 1 [file viruses-17-00952-s001.zip › Supplementary tables 1-4.pdf]

**Table S1** the primers for PCR amplification of the whole genome sequence of GLRaV-4 isolates BSN and FaS

| Primer name | Primer sequence (5′→3′) | Position |
|---|---|---|
| BSN-GLRaV-4-1F | CTACCATCCCCTTCTGAAGCC | 173-193 |
| BSN-GLRaV-4-1R | GATTTCAAGAAGCACTTAGAAGTC | 1458-1435 |
| BSN-GLRaV-4-2F | GTTATTATGTCGGACAGGTGATAG | 1335-1359 |
| BSN-GLRaV-4-2R | CTTTGGCTTGGTTTTGTAGATG | 2733-2712 |
| BSN-GLRaV-4-3F | GACAAGTTCATTGAGGTCTGAC | 2611-2632 |
| BSN-GLRaV-4-3R | ATCAAGACAGATGCTGCAAGAGGGAAGAGTAT | 4041-4010 |
| BSN-GLRaV-4-4F | GCTTGCGGTTTTAAAGTTATCG | 3923-3902 |
| BSN-GLRaV-4-4R | TAACCGTCTCCTTTTCTG | 5298-5281 |
| BSN-GLRaV-4-5F | CTCTTGCGGATTGAACATAGATG | 5179-5201 |
| BSN-GLRaV-4-5R | GGACATGTAACATTCATCCAAGAG | 6598-6575 |
| BSN-GLRaV-4-6F | GAATAAGAAGGTGTTAGTTATGAC | 6493-6516 |
| BSN-GLRaV-4-6R | CAAACTCTTCTAGAGCTTCCG | 7896-7876 |
| BSN-GLRaV-4-7F | GTAAAGGAGGTCTGAACAGAG | 7771-7791 |
| BSN-GLRaV-4-7R | CAGCGTTGAATTTGTTCATGC | 9191-9171 |
| BSN-GLRaV-4-8F | CATACCAACTGTTGTGGGTATAAG | 9064-9087 |
| BSN-GLRaV-4-8R | TTAGGGTCTCCTAAGATCCTGGAG | 10500-10477 |
| BSN-GLRaV-4-9F | AGTCCTGCTGCGAATGAATAC | 10389-10408 |
| BSN-GLRaV-4-9R | CAACATGTAGGCCACATCAGC | 11891-11871 |
| BSN-GLRaV-4-10F | GGTGTGGTTAAAACTGTAGATTTC | 11722-11795 |
| BSN-GLRaV-4-10R | GCCTGACATGGTTTCAGATAG | 11735-11715 |
| BSN-GLRaV-4 5’-outer | CTACCATCCCCTTCTGAAGCC | 193-173 |
| BSN-GLRaV-4 5’-inner | CTGAGAATGCTACGTCCTAGC | 209-229 |
| BSN-GLRaV-4 3’-outer | CTATCTGAAACCATGTCAGGC | 10735-10715 |
| BSN-GLRaV-4 3’-inner | TTTTACGGTGACAGCATG | 13606-13623 |
| FaS-GLRaV-4-1F | AGATTATTTCAAAAAATATTATACTT | 17-42 |
| FaS-GLRaV-4-1R | GTCTTAGAGAACTCTTTCCCTTTG | 1542-1518 |
| FaS-GLRaV-4-2F | AGTGGTGGTGAATGAACCTA | 1369-1389 |
| FaS-GLRaV-4-2R | CATCATCCAATAACTTCTGAACT | 2831-2791 |
| FaS-GLRaV-4-3F | GTTGGTCAATCAGAATGGA | 2674-2693 |
| FaS-GLRaV-4-3R | AGAACCTTCTCTCTTATCCTCTTCGA | 4107-4081 |
| FaS-GLRaV-4-4F | AAGGCTATTGGTCTTAAACCT | 3940-3961 |
| FaS-GLRaV-4-4R | ACAATGATCAAACAAGTTAGGGTT | 5425-5401 |
| FaS-GLRaV-4-5F | CAGTCCCTGGTCCACTTCCTTTG | 5241-5263 |
| FaS-GLRaV-4-5R | ATATTCAACATGCCCATAGTGTC | 6757-6733 |
| FaS-GLRaV-4-6F | CTACATGTCACATGCTGGTC | 6616-6635 |
| FaS-GLRaV-4-6R | TTGCCAGTGACTCCCCACCCAGAGCT | 8106-8081 |
| FaS-GLRaV-4-7F | GACAGGTTAACTTGCGCTC | 7951-7968 |
| FaS-GLRaV-4-7R | TATGAACTGTATTCTGCAGGCACT | 9432-9409 |
| FaS-GLRaV-4-8F | GAGAAAGGTAAAACAAGG | 9299-9316 |
| FaS-GLRaV-4-8R | TTTACTGAACCAGAGACGCCAT | 10792-10771 |

| Primer name | Primer sequence (5′→3′) | Position |
|---|---|---|
| FaS-GLRaV-4-9F | GTGTCGGGAACAGATGAACA | 10608-10627 |
| FaS-GLRaV-4-9R | GAGCAGTTTCTTCAACATTGTC | 12188-12167 |
| FaS-GLRaV-4-10F | CTACTTGATAGGTTCAGGTCA | 12051-12071 |
| FaS-GLRaV-4-10R | GGATCACAGATGCCTGACATGG | 13774-13754 |
| FaS-GLRaV-4 5'-outer | TTTCAAAAAATATTATACTT | 108-89 |
| FaS-GLRaV-4 5'-inner | TTGAGCGCTTTCACCATCCCC | 217-237 |
| FaS-GLRaV-4 3'-outer | GTCAGGCATCTGTGATCCTG | 13776-13757 |
| FaS-GLRaV-4 3'-inner | GTTTTACGGTGATAACATG | 13633-13651 |

**Table S2** the primers used in virus detection to validate the high-throughput sequencing results

| Primer name | Primer sequence (5′→3′) |
|---|---|
| GGVA-F | CAGTATCTTCTGAGGCACGA |
| GGVA-R | GCTAACGACGCTTATGCTGA |
| GFKV-F | CGCTAGGGCTGTGGAAGTATT |
| GFKV-R | CGCTAGGGCTGTGGAAGTATT |
| GRSPaV-F | TGAAGGCTTTAGGGGTTAG |
| GRSPaV-R | TGAAGGCTTTAGGGGTTAG |
| GPGV-F | TGAGATCAACAGTCAGGAGAG |
| GPGV-R | GAAGCCGTGATAGCATTAGTC |
| GLRaV-2F | GGTGATAACCGACGCCTCTA |
| GLRaV-2R | CCTAGCTGACGCAGATTGCT |
| GLRaV-4F | ACATTCTCCACCTTGTGCTTTT |
| GLRaV-4R | CATACAAGCGAGTGCAATTAC |
| GAMaV-F | CTAGCGACGACCGCACTGATC |
| GAMaV-R | GTCGGTGTACGAGATTTGGTC |
| GVB-F | ATCAGCAAACACGCTTGAACCG |
| GVB-R | GTGCTAAGAACGTCTTCACAGC |
| GRVFV-F | GGAATGGCTGAAGCAACGTC |
| GRVFV-R | GCTTCTGCGCATTTCCAGTC |
| GSyV-1-1F | GACACGACTCGTTATGAGCAG |
| GSyV-1-1R | CACTTGGCGAATGTGGAGTTG |
| GYSVd1-F | TCTCCGGATCTTCTTGCTTG |
| GYSVd1-R | CCTCTTAGCGGGGGTTCCGG |
| GYSVd2-F | CGAAGCCGGTTTGAGGCCCG |
| GYSVd2-R | AGTCCGAGGACCTTTTCTAG |
| HSVd-F | CTGGGGAATTCTCGAGTTGC |
| HSVd-R | AGGGGCTCAAGAGAGGATCC |

**Table S3** the primers used to construct the vector

| Primer name | Primer sequence (5′→3′) |
|---|---|
| AD-F | GGATCCATCGAGCTCGAGCTGCA |
| AD-R | GAATTCACTGGCCTCCATGGCCAT |
| BD-F | GGATCCGTCGACCTGCAGCG |
| BD-R | GAATTCGGCCTCCATGGCCATA |
| AD-RdRp-F | atggccatggaggccagtgaattcATGTTTAGCATGTATGTCATGACTG |
| AD-RdRp-R | ctgcagctcgagctcgatggatccTTACTTACCCTTGTCAATCACCTT |
| AD-p5-F | atggccatggaggccagtgaattcATGTTGGATTTGTTTTCACAATT |
| AD-p5-R | ctgcagctcgagctcgatggatccTTATGCCGGTGGTGCTG |
| AD-HSP70h-F | atggccatggaggccagtgaattcATGGAGGTGGGTATAGATTTTG |
| AD-HSP70h-R | ctgcagctcgagctcgatggatccCTAGTCGCAGACAATGCCAT |
| AD-HSP90h-F | atggccatggaggccagtgaattcATGGCATTGTCTGCGACTAG |
| AD-HSP90h-R | ctgcagctcgagctcgatggatccCTTTGAATGTCCTTAACAGATCC |
| AD-cp-F | atggccatggaggccagtgaattcATGGCAAGTGTCGGTAATAAC |
| AD-cp-R | ctgcagctcgagctcgatggatccTCATCTTCTATTGCCCAAGAA |
| AD-p23-F | atggccatggaggccagtgaattcATGGAGGTTGTTCTTTGCTTA |
| AD-p23-R | ctgcagctcgagctcgatggatccTCATCTTCTATTGCCCAAGAA |
| BD-RdRp-F | catatggccatggaggccgaattcATGTTTAGCATGTATGTCATGACTG |
| BD-RdRp-R | cggccgctgcaggtcgacggatccTTACTTACCCTTGTCAATCACCTT |
| BD-p5-F | catatggccatggaggccgaattcATGTTGGATTTGTTTTCACAATT |
| BD-p5-R | cggccgctgcaggtcgacggatccTTATGCCGGTGGTGCTG |
| BD-HSP70h-f | catatggccatggaggccgaattcATGGAGGTGGGTATAGATTTTG |
| BD-HSP70h-R | cggccgctgcaggtcgacggatccCTAGTCGCAGACAATGCCAT |
| BD-HSP90h-F | catatggccatggaggccgaattcATGGCATTGTCTGCGACTAG |
| BD-HSP90h-R | cggccgctgcaggtcgacggatccCTTTGAATGTCCTTAACAGATCC |
| BD-cp-F | catatggccatggaggccgaattcATGGCAAGTGTCGGTAATAAC |
| BD-cp-R | cggccgctgcaggtcgacggatccTCATCTTCTATTGCCCAAGAA |
| BD-p23-F | catatggccatggaggccgaattcATGGAGGTTGTTCTTTGCTTA |
| BD-p23-R | cggccgctgcaggtcgacggatccTCATCTTCTATTGCCCAAGAA |
| NYFP-F | TGACTAGAGTCGACCTGCAGAG |
| NYFP-R | GATCCGCCGGCGTCCTCGATGT |
| CYFP-F | GGAGGAGTTTGATAAGGTGTTCTC |
| CYFP-R | GTACCTGTAATTGTAAATAGTAAT |
| NYFP3-F | acaagtttgtacaaaaaagcaggcATGGAGGTGGGTATAGATTTTG |
| NYFP3-R | caccactttgtacaagaaagctgTGTCGCAGACAATGCCATGTTT |
| NYFP5-F | acaagtttgtacaaaaaagcaggctATGGCAAGTGTCGGTAATAAC |
| NYFP5-R | caccactttgtacaagaaagctgTTCTTCTATTGCCCAAGAAAATGT |
| CYFP6-F | acaagtttgtacaaaaaagcaggctccATGGAGGTTGTTCTTTGCTTA |
| CYFP6-R | caccactttgtacaagaaagctgTCGATAGAGAGAAGGTTAACCACC |

**Table S4** Viruses species analyses of high-throughput sequencing data

| Sample | Virus/Viroid | Reference access number | Reference length | Coverage (%) | conitgs counts | Depth | Depth (Norm) | %Identity | %Iden Max | %Iden Min |
|---|---|---|---|---|---|---|---|---|---|---|
| Baisainie | GLRaV-4 | KY821095 | 13857 | 99.1 | 1 | 56.6 | 0.9 | 86.17 | 86.17 | 86.17 |
| | GPGV | FR877530 | 7259 | 97.9 | 5 | 379.6 | 5.9 | 95.9 | 96.6 | 95.32 |
| | GFkV | AJ309022 | 7564 | 45.1 | 20 | 2547.1 | 39.5 | 97.37 | 100 | 90.3 |
| | GRSPaV | MG938328 | 8726 | 99.8 | 30 | 710.2 | 11.1 | 98 | 99.38 | 91.94 |
| | GAMaV | KX354202 | 6719 | 88.8 | 3 | 82.7 | 1.3 | 92.91 | 94.59 | 92.37 |
| | GSyV-1 | KT037017 | 6440 | 94.6 | 4 | 71.3 | 1.1 | 86.44 | 91.14 | 83.3 |
| | HSVd | AB054614 | 301 | 100 | 5 | 140.6 | 2.2 | 95.2 | 95.83 | 94.12 |
| | GYSVd1 | AB028466 | 368 | 100 | 3 | 130.7 | 2.0 | 97.42 | 97.95 | 97.10 |
| Fantasy Seedless | GLRaV-4 | KY821095 | 13857 | 100 | 25 | 472,6 | 5.4 | 99.26 | 100 | 97.67 |
| | GLRaV-2 | AY881628 | 16494 | 99.6 | 35 | 872.2 | 10.0 | 99.15 | 99.72 | 96.69 |
| | GVB | KF700375 | 7610 | 98.6 | 8 | 310.6 | 3.5 | 98.95 | 99.66 | 90.07 |
| | GPGV | MN458446 | 7247 | 99.3 | 28 | 877.4 | 10.1 | 97.16 | 99.52 | 95.60 |
| | GFkV | AJ309022 | 7564 | 22.5 | 13 | 865.9 | 10.0 | 95.01 | 98.10 | 85.06 |
| | GRVFV | MN974275 | 6730 | 18.4 | 10 | 18.5 | 0.2 | 83.90 | 87.23 | 81.22 |
| | GRSPaV | KR054734 | 8753 | 99.3 | 18 | 694.8 | 8.0 | 99.02 | 99.53 | 97.97 |
| | HSVd | MF576417 | 303 | 100 | 3 | 231.1 | 2.7 | 97.01 | 98.19 | 93.02 |
| | GYSVd1 | AB028466 | 368 | 100 | 7 | 68.5 | 0.8 | 96.85 | 97.62 | 96.33 |
| | GYSVd2 | MT338566 | 379 | 97.4 | 3 | 188.4 | 2.2 | 97.83 | 98.33 | 97.55 |
